# Changes in Biochemical Properties and Activity of Trypsin-like Protease (*Litopenaeus vannamei*) Treated by Atmospheric Cold Plasma (ACP)

**DOI:** 10.3390/foods11091277

**Published:** 2022-04-28

**Authors:** Lingling Tang, Shaimaa Hatab, Jinhong Yan, Wenhua Miao, Bhoke Marwa Nyaisaba, Xinyue Piao, Bin Zheng, Shanggui Deng

**Affiliations:** 1 Department of Food Science and Pharmaceutics, Zhejiang Ocean University, Zhoushan 316022, China; s19083200002@zjou.edu.cn (L.T.); mzx818123@163.com (J.Y.); nyaisabamarwa@gmail.com (B.M.N.); z20095135039@zjou.edu.cn (X.P.); zhengbin@zjou.edu.cn (B.Z.); dengshanggui@163.com (S.D.); 2 Faculty of Environmental Agricultural Science, Arish University, North Sinai 45516, Egypt; shimaa.reda@hu.edu.eg; 3 Faculty of Organic Agriculture, Heliopolis University, Cairo 2834, Egypt

**Keywords:** trypsin-like protease, atmospheric cold plasma, enzymatic properties, inhibition

## Abstract

The changes in the functional properties of trypsin from shrimps (*Litopenaeus vannamei*) after, Atmospheric Cold Plasma (ACP) treatments, have been evaluated in terms of enzyme inactivation, surface hydrophobicity, secondary structure, fluorescence intensity, and particle size distribution. Different exposure voltages of 10, 20, 30, 40, and 50 kV at various treatment times (1, 2, 3, and 4 min) have been employed, in a separate assay. The results showed that trypsin-like protease activity decreased (by about 50%), and the kinetic constants K_m_ value increased, while the k_cat_ value decreased. Surface hydrophobicity and fluorescence intensity revealed a significant increase compared to the control sample. A high degree of protein degradation has been noticed by SDS-PAGE analysis. In addition, circular dichroism indicated that random coil and α-helix contents declined while β-turn and β-sheet contents have raised. A sharp drop in the particle size was observed with increasing the treatment voltage from 0 to 40 kV for 4 min, and the corresponding peak reached the minimum of 531.2 nm. Summing up the results, it can be concluded that the ACP technique effectively affects the activity of trypsin-like protease, which in terms enhances the quality of dietary protein.

## 1. Introduction

Seafood is considered nutritious food rich in minerals, vitamins, and essential amino acids that are important for the human diet. The increase in consumer awareness and the rise in the human population resulted in a growth in demand for seafood products [1]. The growing demand could be covered by maintaining the quality of these products and reducing the waste rate. Endogenous proteases are among the main causes of post-mortem degradation of seafood muscles leading to muscular disorganization and deterioration of myofibres which is called “Softening” [2]. Trypsin, as an example of serine proteases, is mostly assisted living beings in the digestion of proteins and promotion of absorption and production of other active digestive enzymes [3]. In *Litopenaeus vannamei* species, trypsin-like protease has a strong effect on the degradation of myofibrillar and collagen. Where trypsin-like protease plays the main role in softening the muscle of *Litopenaeus vannamei* [4], it is considered the key autolytic enzyme as well in the head of *Litopenaeus vannamei*, that which hydrolyzes muscle proteins [5]. After the catching of *Litopenaeus vannamei*, trypsin-like protease gradually migrates and diffuses into the muscle tissue, resulting in the degradation of the whole-body muscle [4]. For instance, muscle autolysis caused by trypsin-like protease leads to a separation of shrimp shells and flesh, the shedding of shrimp heads, softening of the muscle, and other phenomena which in turn reduce the shrimp freshness notably [6]. During the autolysis process, the muscle protein of shrimp is decomposed and a series of intermediate products such as peptides, amino acids, and soluble nitrogen compounds are produced [7]. These intermediate products promote the growth and reproduction of spoilage micro-organisms which in turn accelerate the further decomposition of the muscle causing the production of ammonia, histamine, trimethylamine, hydrogen sulfide, indoles, and other small molecular substances, which induce off-odor as an indicator of shrimp spoilage [6]. Therefore, to maintain the quality of *Litopenaeus vannamei*, extend its shelf life, and minimize muscle degradation, inhibiting the activity of proteases is essential. To alleviate this problem, protease inhibitors (PIs) have been used to block the active sites or allosteric sites of proteases or their zymogens to inactivate these enzymes [8]. Bijina et al. [9] stated that the protein degradation of shrimp (*Penaeus monodon*) significantly decreased after treatment with 0.2 mg/mL protease inhibitor isolated from *Moringa oleifera*. It has been found that the trypsin inhibitor (TI) obtained from dried seed powder effectively inhibits the proteolytic enzymes during surimi gelation, where 30 g/L of TI can suppress the degradation of sarcoplasm by 76% [10]. However, in some cases, natural and synthetic inhibitors can lead to adverse consequences. Where some enzyme inhibitors have antinutritive properties, for example, trypsin inhibitors could hinder the activity of protein-digesting enzymes in the digestive tract and therefore reduce the ability of the body to utilize food protein [11].

In addition to chemical methods for trypsin inhibition, some physical approaches such as atmospheric cold plasma (ACP) have been studied as a potential alternative for enzymes inhibitors. Atmospheric cold plasma (ACP), non-thermal processing technology, has been investigated for its ability to inhibit enzyme activity, change the molecular structure, and maintain the quality properties of food. Dielectric Barrier Discharge (DBD) is widely used to generate atmospheric pressure, and the active particles required for the chemical reaction can be obtained at room temperature. The device is relatively simple, and the reaction conditions are easy to control and operate. In the ACP device, the plasma is evenly distributed throughout the whole dielectric space, at this time, a series of complex reactions occur between the active substances generated by the discharge and the sample. Under high voltage, plasma can be ionized by neutral gas to generate negatively and positively charged ions, free electrons, free radicals, excited or unexcited molecules and atoms, and ultraviolet photons [12]. ACP operation involves the production of active substances, for instance, reactive nitrogen (RNS) and reactive oxygen species (ROS) [13].

Recent studies showed auspicious results for using ACP to inhibit enzyme activity such as polyphenol oxidase (PPO) in apples and mushrooms [14], peroxidase (POD) in tomatoes [15], and endogenous protease in squid [16]. However, there is still considerable ambiguity regarding the effect of ACP on trypsin-like protease activity in *Litopenaeus vannamei*. Therefore, the effect of ACP on enzyme structure, stability as well as other physicochemical properties of trypsin-like protease extracted from shrimps (*Litopenaeus vannamei*), have been investigated in this research. Different voltage levels (10, 20, 30, 40, and 50 kV) for different treatment times (1, 2, 3, and 4 min) as factors influencing the efficiency of ACP treatment, have been considered.

## 2. Materials and Methods

### 2.1. Trypsin Extraction from Shrimp

#### 2.1.1. Preparation of Hepatopancreas Extract

Shrimps (*Litopenaeus vannamei*) were obtained from the fish market at Zhoushan, Zhejiang Province, China. Shrimps were about 6 g in weight and 24 cm long. Plastic bags filled with crushed ice were used for samples transportation (within 30 min) to the Food Science laboratory at Zhejiang Ocean University. Upon arrival, the samples were shocked with cold water (0 °C) and the hepatopancreas were collected and powdered in liquid nitrogen. The hepatopancreas extract was prepared using the method of Khantaphant and Benjakul, ref. [17] with minor modifications. Hepatopancreas were homogenized with six volumes of 20 mM Tris–HCl buffer (pH 8.0) containing 5 mM CaCl_2_ for 3 min using an IKA homogenizer. The homogenate was centrifuged at 8000∗ *g* for 30 min at 4 °C. The supernatant obtained was filtered using a Whatman filter paper No. 1. The obtained filtrate was referred to as hepatopancreas extract.

#### 2.1.2. Ammonium Sulfate Fraction Preparation

Preparation of ammonium sulfate fraction was done as described by Bassompierre et al. [18] with slight modifications. Hepatopancreas extract was exposed to 30–60% saturated ammonium sulfate precipitation. The mixture was stirred for 30 min at 4 °C followed by the centrifugation at 8000∗ *g* for 30 min at 4 °C to obtain a pellet which was dissolved in a minimum volume of 20 mM Tris–HCl buffer (pH 8.0) containing 5 mM CaCl_2_ and dialyzed against the same buffer at 4 °C overnight. The dialysate obtained was considered as ammonium sulfate fraction.

#### 2.1.3. Purification of Trypsin

The dialysate from the Ammonium sulfate fraction was then applied to a Q-Sepharose Fast Flow anion exchange column (3.5 cm × 20 cm), which was previously equilibrated with the dialysis buffer. The sample was loaded onto the column at a flow rate of 2 mL/min. After the un-adsorbed protein was thoroughly washed with the initial buffer solution, the column was eluted with a linear gradient of 0.3 and 1 M NaCl in 20 mM Tris–HCl buffer (pH 8.0) at a flow rate of 2 mL/min, and each component was collected. BAPNA (Na-benzoyl-DL-arginine-nitro amide hydrochloride) was used as a substrate to determine the enzyme activity of each tube, and the high activity components were collected. The active components were fully dialysed against a buffer of 20 mM Tris–HCl buffer (pH 8.0) and freeze-dried to obtain purified trypsin.

### 2.2. Evaluation of Trypsin Activity

Trypsin activity was measured according to the method described by Senphan et al. [6] using BAPNA as a substrate. An appropriately diluted enzyme (50 µL) was added to 2.2 mL of 20 mM Tris–HCl buffer (pH 8.0). The reaction was immediately initiated by the addition of 150 µL of 10 mM substrate and incubated at 37 °C for 20 min. To stop the reaction, 200 µL of the stopping agent (cold 10% (*w*/*v*) trichloroacetic acid) was added. The absorbance value was measured at 410 nm by UV-vis spectrophotometer (U-2600, Hitachi, Tokyo, Japan). A blank solution was prepared in the same way, with the exception that the enzyme sample was added after the addition of 10% trichloroacetic acid. Each sample was repeated 3 times. The unit of activity (U) of trypsin is defined as the amount of enzyme required to convert 1 μmol of substrate per minute.

### 2.3. Determination of Protein Concentration

Protein concentration was determined according to the method of Lowry et al. [19] using bovine serum albumin as standard. Each sample was repeated 3 times.

### 2.4. Sodium Dodecyl Sulfate-Polyacrylamide Gel Electrophoresis (SDS-PAGE)

SDS-PAGE was carried out as described by Laemmli [20]. Trypsin samples were dissolved in protein buffer (containing 4% SDS, 20% glycerol, 0.02% bromophenol blue, 0.125 mol/L Tris-HCl buffer, pH 6.8) at a ratio of 1:3 (trypsin sample: protein buffer), then the mixture was heated in a water bath at 100 °C for 3 min. Samples were analyzed in an electrophoresis system, using 4% stacking gel and 10% separating gel, respectively. The thickness of the electrophoresis gel plate was 1 mm. The amount of marker and sample loaded were 5 μL. The system was run at 80 V until the sample and protein marker aligned, then the power was increased to 120 V for 1 h. After that, the gel was dyed carefully with 0.1% (*w*/*v*) Coomassie brilliant blue in 50% (*v*/*v*) methanol and 7.5% (*v*/*v*) acetic acid then decolorized in deionized water containing 7.5% (*v*/*v*) acetic acid and 5% (*v*/*v*) methanol. The molecular weight (MW) of each protein band was assessed using a distance of protein migration in comparison to the MW of the protein marker. Solarbio Science & Technology Co., Ltd., Beijing, China. Protein marker PR1910 (11 kD–180 kD) was used as a protein marker.

### 2.5. Sample Preparation and Atmospheric Cold Plasma Treatment

Trypsin solution was prepared according to the method described by Yoshida, Bae [21] with slight modifications. An appropriate amount of trypsin was dissolved in 20 mM Tris–HCl buffer (pH 8.0) to reach a final concentration of 1 mg/mL. Ten mL of the prepared trypsin solution was placed in a petri dish with a diameter of 89 mm and thickness of 2 mm and then the dish was placed in DBD plasma, between two parallel rounded aluminum plates with an outer surface of 155 mm. Dielectric Barrier Discharge (DBD) plasma (Phenix BK130/3 AC Test Set 600 series processor, Phenix Technologies, Inc., Accident, MD, USA) has been used in this study, where the reaction conditions are easy to control, operate, and the plasma generated in discharge space is relatively homogenous. DBD in the form of polystyrene boards (2 mm thickness) in each end separated both the electrodes and the sample. The distance between both electrodes was 75 mm. A high voltage transformer was used to deliver the necessary energy required for the generation of plasma from atmospheric gas. The samples were then subjected to 10, 20, 30, 40, and 50 kV voltages for different treatment times 1, 2, 3, and 4 min, separately. Trypsin solution with no treatment of plasma was included as a control.

### 2.6. Kinetic Studies

To determine the respective enzymatic activities, the prepared trypsin with a final concentration of 1 mg/mL was allowed to react with different concentrations (0.4–2.4 mM) of various BAPNA-substrates at 37 °C for 20 min. Kinetic parameters including the maximum velocity (V_max_) and Michaelis-Menten constant (K_m_) were evaluated based on the Lineweaver-Burk plots (Lineweaver & Burk, 1934). Values of turnover number (k_cat_) were calculated from the following equation: V_max_/[E] = k_cat_, where [E] refers to the active enzyme concentration. Each sample was repeated 3 times.

### 2.7. The Effect of ACP on Temperature and pH Profile of Trypsin

The method described by Sriket, Benjakul [5] was used to determine the changes in temperature and pH of trypsin solution after treatment with ACP. In this study, the temperature of samples was measured using a normal thermometer, and a pH meter (Sartorius PB-10, Germany) was utilized to determine the changes in the pH. Trypsin solutions without ACP treatment were included as a control. Each sample was repeated 3 times.

### 2.8. Assay Surface Hydrophobicity

The binding of bromophenol blue (BPB) methods has been used to assay the surface hydrophobicity of trypsin [22]. Briefly, 2 mL of trypsin solution (1 mg/mL) was dissolved in 20 mM Tris-HCl buffer (pH 8.0), then mixed with 20 µL of BPB (1 mg/mL), followed by incubation for 10 min at room temperature with a short pulse of vortex every 10 min. After incubation, the samples were centrifuged in 2000× *g* for 15 min at 4 °C. Finally, the obtained supernatant was diluted 10 times with Tris-HCl buffer in a clean centrifuge tube, then the absorbance was measured by Hitachi U-2000 UV-Vis spectrophotometer (Tokyo, Japan) at 595 nm. Samples without trypsin solution (20 µL of BPB with 2 mL of Tris-HCl buffer) were included as a control. The experiment has been performed in triplicate.

The following formula has been used to calculate the quantity of bound BPB (as a hydrophobicity index):

BPB bound (µg) = 200 µg × (OD Control − OD Sample)/(OD Control)

### 2.9. Assay the Total Content of Sulfhydryl

The influence of ACP treatment on the sulfhydryl (SH) content has been detected as mentioned by Nikoo, Benjakul [23] with slight modifications. One mL trypsin solution (1 mg/mL) was dissolved in 20 mM Tris-HCl buffer (pH 8.0), then 8 mL of dissociating buffer (5.2 g Tris-HCl, 3.45 g glycine, 240 g urea, 0.6 g EDTA in 500 mL distilled water, pH 8.0) and 0.5 mL of DTNB (4 mg/mL of 5, 5-dithiobis-(2-nitrobenzoic acid)) were added. The mixture was incubated in a dark place at room temperature for 10 min. After incubation, the absorbance was read at 412 nm using a Hitachi U-2000 UV-Vis spectrophotometer (Tokyo, Japan). An 8 mL mixture of the dissociating buffer and 0.5 mL DTNB without trypsin solution was considered as a control and handled in the same pattern. Calculations for SH concentration in the samples involved a molar absorbance coefficient of 13,600 (mol/L)^−1^ cm^−1^, and results were expressed as nmol total SH/mg of protein.

### 2.10. Determination of Circular Dichroism

The method given by Farasat, Arjmand [24] was applied to evaluate the impact of ACP on the secondary structures of trypsin, with slight changes. One hundred µL trypsin solutions (1 mg/mL, pH 8.0) were treated by ACP at 10, 20, 30, 40, and 50 kV for 4 min. The ACP treated samples as well as untreated samples (control) were put in a 1 cm length quartz cuvette and analyzed using a spectropolarimeter (USA) at 25 °C with a spectra range of 180–260 nm to estimate the content (%) of α-helix, β-sheet, β-turn, and random coils.

### 2.11. Intrinsic Fluorescence Spectroscopy

A wavelength of 280 nm was used to excite the intrinsic tryptophan fluorescence of trypsin, and the fluorescence emission spectrum was observed in the range of 300–400 nm [25]. The deionized water was used to dilute the trypsin solution to a concentration of 0.2 mg/mL, where the protein with low concentration is typically constant in terms of total absorbance of the solution, and fluorescence intensity is correlated to the protein concentration [26]. Then, the solution’s intrinsic fluorescence intensity was determined using a Hitachi F-7000 fluorospectrophotometer (Hitachi Ltd., Tokyo, Japan), at room temperature. The following parameters were used: excitation wavelength of 280 nm, slit for excitation of 5.0 nm; emission wavelength of 300–380 nm, slit 5 nm, and scanning speed 60 nm/min.

### 2.12. Determination of Particle Size

The PALS laser particle size analyzer (Nano. ZS90, Malvern Instruments Ltd., Malvern, UK) was applied to determine the particle size distribution of trypsin [27]. The trypsin was added to a protein solution with a concentration of 10 mg/mL using 20 mmol/L Tris–HCl buffer (pH 8.0), and 1 ml was taken in a colorimetric dish and measured at room temperature. Particle size was automatically obtained using Malvern standard operating procedure software, and the experiment was repeated three times, with representative results selected for inclusion in the paper.

### 2.13. Statistical Analysis

Each experiment was performed in triplicates (*n* = 3). Data were presented as mean ± SD and statistical analysis was done using SPSS software (SPSS Version 20.0, SPSS Inc., Chicago, IL, USA) based on a one-way analysis of variance (ANOVA), and the differences between the mean of the data were compared using Duncan’s multiple range test at significance level *p* < 0.05.

## 3. Results and Discussion

### 3.1. Purification of Trypsin from Shrimps (Litopenaeus vannamei)

**Figure 1:**
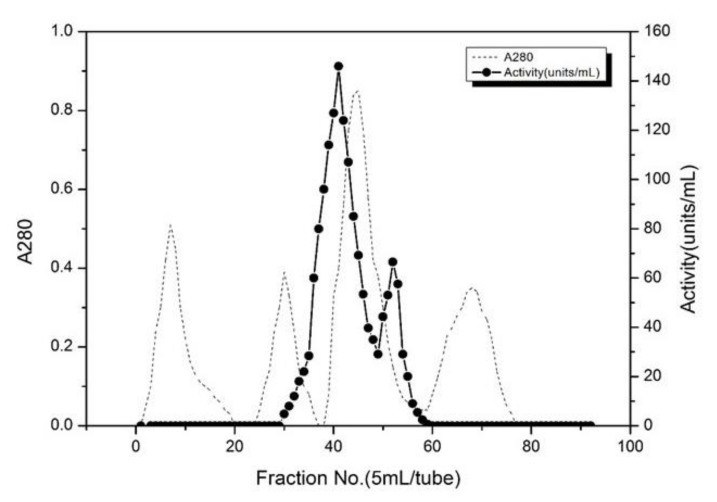
Elution profile of trypsin extracted from *Litopenaeus vannamei*, on Q-Sepharose anion exchange column. The graph shows a typical result of several repeated experiments.

Table 1 shows the purification results of trypsin. The trypsin was characterized by separating the crude enzyme using ammonium sulfate fractionation and a Q-Sepharose Fast Flow anion exchange column. Ammonium sulfate precipitation with 30–60% saturation was effective in the separation of trypsin from other proteins in the crude enzyme, resulting in a significant increase in purity by 10.0-fold and the obtained yield was about 82.3%. Ammonium sulfate precipitation is an effective method for the purification of the crude extract from other proteins [17]. A purity of 84.3-fold was observed after using the Q-Sepharose anion exchange column and a yield of 24.9% was obtained. The contaminating proteins, specifically those that bind with trypsin have been removed by using Q-Sepharose F F anion exchange chromatography [28,29]. During the elution process through Q-Sepharose F F anion exchange chromatography, four peaks were observed (Figure 1), the activity of the enzyme solution corresponding to the four peaks was measured, and only the enzyme solution at the third peak (30–60 tube) was active, the third peak showed the highest activity. Hence the third peak fraction was collected and referred to as highly purified trypsin. Poonsin, et al. [30] also purified anionic trypsin from the spleen of albacore tuna (*Thunnus alalunga*) by using a fast flow anion exchange column.

### 3.2. Purity and Molecular Weight of Trypsin from Shrimps (Litopenaeus vannamei)

**Figure 2:**
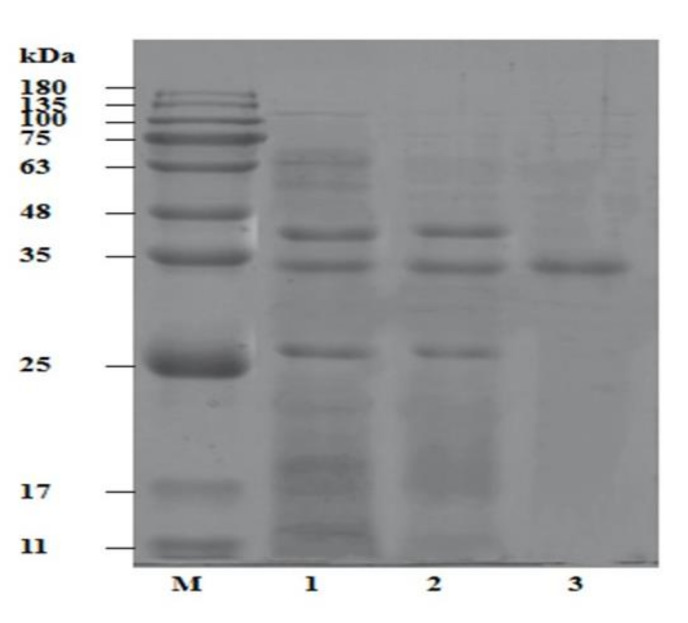
SDS-PAGE of the trypsin from *Litopenaeus vannamei*. Lanes: M, protein marker; 1, crude enzyme; 2, ammonium sulfate fractional precipitation; and 3, Q-Sepharose anion chromatography column. Representative results from repeated experiments were selected.

As indicated in SDS–PAGE profile (Figure 2), the extract contained several protein bands with high MW and low MW signifying sarcoplasmic proteins and enzymes. Large MW proteins were removed after the fractionated precipitation of ammonium sulfate, three bands with approximately MW of 47 kDa, 33 kDa, and 24 kDa. Moreover, after being purified using a Q-Sepharose anion chromatography column, a single protein band with approximately MW of 33 kDa was observed, the bands with MW of 47 kDa and 24 kDa were further removed. The outcome indicated that the Q-Sepharose anion chromatography column was able to remove contaminants from the extract and purify trypsin. Additionally, The obtained MW of the trypsin is consistent with that obtained by Honjo et al. [31] and Perera et al. [32] who observed trypsin with MW of 36 kDa from hepatopancreas of shrimp (*Penaeus indicus*) and MW of 35–36 kDa from lobster hepatopancreas.

### 3.3. Effect of ACP on the Activity of Trypsin

**Figure 3:**
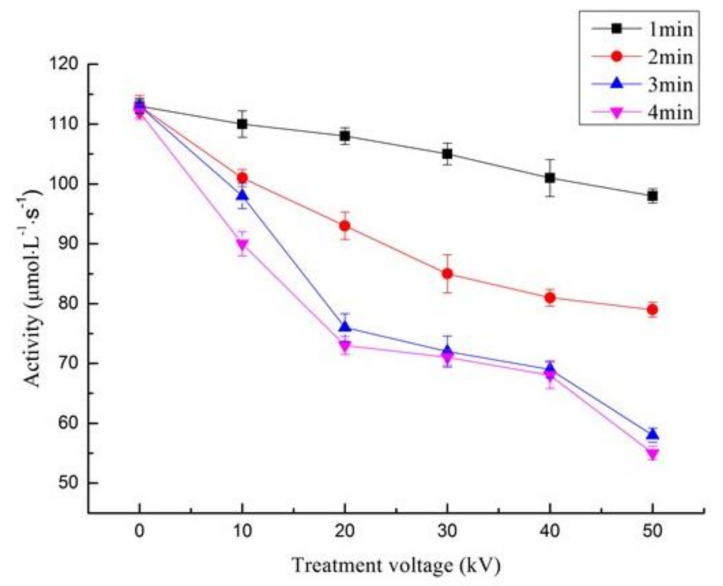
Impact of ACP treatment at a different time and voltages on the activity of trypsin (mean ± SD, *n* = 3). Control (0 min) refers to samples without ACP treatment.

Enzymes are proteins composed of amino acid polymers in which their functionality is determined through their specific three-dimensional structures. Thus, changing the natural structural format would inhibit the enzymatic activity. Previous studies have shown that ACP can inhibit endogenous proteases in aquatic products such as squid [16], hairtail fish, and polyphenol oxidase in shrimp heads. Many researchers indicate remarkable changes in the protein structures, as well as the peptide bonds, occur after ACP treatment [33]. The activity of trypsin-like protease before and after ACP treatment is presented in Figure 3. The inhibition of trypsin-like protease activity by ACP strongly depends on treatment time and voltage intensity. Compared with the control group, the trypsin activity decreased significantly (*p* < 0.05) with the extension of treatment time and stabilizing the voltage intensity. Similarly, an inhibition in trypsin activity was observed with increasing the voltage intensity and fixing the treatment time. The activity of the enzyme reduced sharply after 4 min of ACP treatment at 50 kV, where the trypsin activity decreased from 114 μmol·L^−1^·s^−1^ in the control sample to 56 μmol·L^−1^·s^−1^ in the ACP treated samples. These outcomes are in line with Choi, et al. [34] who indicated that the treatment voltage and exposure time are crucial factors that influence the changes in protein structure, which in turn affect enzyme performance. It has been pointed out that dielectric barrier discharge (DBD) produces a high concentration of active species such as ozone and active nitrogen which react with protein resulting in structural changes and subsequent inhibition of enzymatic activity. Moreover, Pankaj, et al. [15] reported a decrease in the activity of pectinase in fresh-cut apples and tomato peroxidase respectively with increasing ACP treatment time.

### 3.4. Kinetic Study

The enzyme kinetic constants can reflect the affinity of the enzyme to the substrate and the catalytic efficiency of the enzyme. The effect of ACP treatment on trypsin kinetic constants (K_m_ and k_cat_) was determined by the Lineweaver-Burk (Table 2). K_m_ and k_cat_ of trypsin without ACP treatment were 4.16 ± 0.01 mM and 25.64 ± 0.16 s^−1^ respectively, but after being exposed to ACP with the increase of treatment time, the K_m_ value gradually increased to 5.04 ± 0.20 mM, while the k_cat_ value decreased to 18.75 ± 0.09 s^−1^ after 4 min of treatment. Moreover, the catalytic efficiency (k_cat_/K_m_) of trypsin decreased from 6.16 ± 0.08 s^−1^ mM^−1^ to 3.72 ± 0.19 s^−1^ mM^−1^ after 4 min of ACP treatment. The increase of K_m_ value reflected the decrease of the affinity of trypsin-like protease to BAPNA substrate, while the decrease in k_cat_ value indicated the decrease in the catalytic capacity of trypsin-like protease. Li, et al. [35] illustrated that a large number of active oxidizes such as ROS and RNS are produced during ACP treatment, which in turn cause oxidation of amino acid side chains and protein backbones resulting in consequential oxidative damage or cross-linking of proteins. This change of kinetic constants was likely caused by the modifications of the binding site between trypsin and substrate. Our results of a kinetic study are coincident with that reported by Ali, et al. [36] which revealed the change in a kinetic constant of mushroom tyrosinase when treated with atmospheric pressure plasma jet (APPJ).

### 3.5. Effect of ACP on Temperature and pH of Trypsin

**Figure 4:**
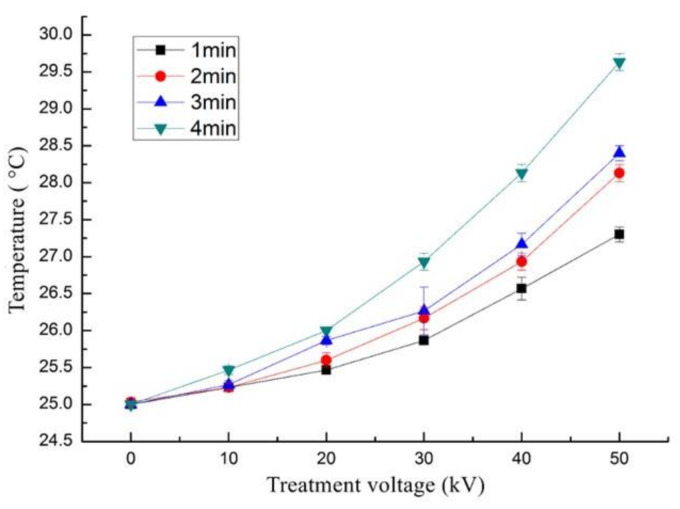
Effect of different ACP treatment voltage and time on the temperature of trypsin (mean ± SD, *n* = 3). Control (0 min) refers to samples without ACP treatment.

Puac, et al. [37] reported that the measurement of pH and temperature after plasma treatment is necessary as the enzyme activity is greatly influenced by pH and temperature factors. The temperature change in trypsin treated with ACP is displayed in Figure 4. Compared with the control group, a slight increase in the temperature of trypsin was observed after ACP treatment, where the highest temperature obtained was 30 °C when the samples were treated at 50 kV for 4 min. The recorded temperature was not sufficient to inactivate the activity of trypsin, where it was reported that trypsin activity starts to decrease when the temperatures exceed 60 °C [29].

Furthermore, the results in Table 3 show that the pH of trypsin did not change significantly after ACP treatment. Our results concur with the outcomes stated by Segat, et al. [38] that the pH of alkaline phosphate was maintained at 6.8 after ACP treatment. Moreover, the pH of squid did not show significant change after ACP treatment as reported in previous studies [35]. Trypsin activity increases at alkaline pH, with optimal pH ranging from 7 to 10 [39]. With the fact that the temperature and pH were insufficient to induce inactivation of the enzyme, thus their effect can be neglected.

### 3.6. Effect of ACP on the Surface Hydrophobicity of Trypsin

**Figure 5:**
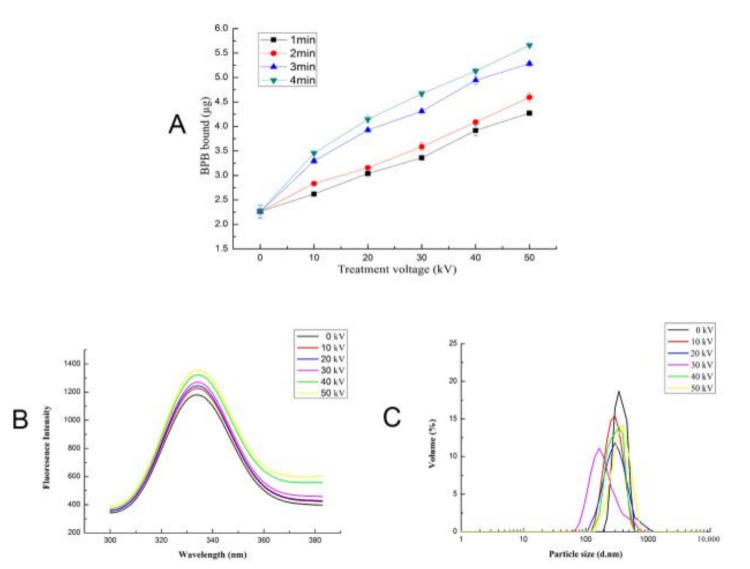
Effect of different ACP treatment voltage and time on surface hydrophobicity (**A**), fluorescence intensity (**B**), and particle size distribution (**C**) of trypsin (mean ± SD, *n* = 3). Control (0 min) refers to samples without ACP treatment.

Surface hydrophobicity indicates the exposure degree of hydrophobic groups within protein molecules. The more hydrophobic groups are exposed, the greater the surface hydrophobicity will be. Bromophenol blue has the function of binding hydrophobic groups, so the surface hydrophobicity is expressed by the amount of exposed hydrophobic amino acid residues that can be bound by bromophenol blue. As depicted in Figure 5A, the surface hydrophobicity of the trypsin solution treated with ACP increased significantly (*p* < 0.05) with the respective increase of voltage and treatment time. The binding amount of bromophenol blue increased from approximately 2.2 µg in control samples to approximately 5.7 µg in the ACP treated samples at 50 kV for 4 min. Surface hydrophobicity is a surface-related property of proteins, which is correlated with the conformational change of protein in its original form. The hydrophobic amino acid residues are situated internally in the protein molecules, and thus the accretion in surface hydrophobicity is due to oxidation, accompanied by exposure to hydrophobic amino acids such as phenylalanine and tryptophan [40]. Trypsin belongs to the basic protein family. Under acidic conditions, due to charge changes at the active site, the enzyme cannot effectively bind to the substrate [41]. Surface hydrophobicity increased in the Silver carp protein subject to the ACP system with the oxide O_3_. Our results came to an end that treatment of trypsin by ACP causes modification of the trypsin conformation to some extent, thus exposing the hydrophobic group.

### 3.7. Effect of ACP on the Total Sulfhydryl of Trypsin

The conformational change of the polypeptide chain, such as embedding and exposure, can be reflected in the change of the sulfhydryl group content to some extent. As illustrated in Table 4, there was no significant difference in total sulfhydryl content between the control and ACP-treated trypsin samples. The sulfhydryl group in proteins mainly refers to the group of a cysteine residue of protein or polypeptide. The unavailability of significant differences could be subjected to the fact that the formation of the disulfide bond in trypsin takes place through stable intermediates with a native conformation composed of one to two native disulfide bonds hence no significant quantity of disulfide intermediate was observed [42]. However, some of the protein undergoes oxidative folding via heterogeneous intermediate and the reductive unfolding of the protein follows a pathway similar to that of the folding [43]. It is worth mentioning that some earlier reports suggested that ACP treatment induces the reduction of the total sulfhydryl group content of the samples [16]. These results are in contrast with our findings, which may be caused by different treatment conditions and different types of enzymes tested.

### 3.8. Effect of ACP on the Fluorescence Intensity of Trypsin

When the protein is at the excitation wavelength of about 280 nm, the endogenous fluorescence comes from tryptophan (Trp) and tyrosine (Tyr) residues, and its endogenous fluorescence is very sensitive to the environment [44]. Therefore, the change of endogenous fluorescence can reflect whether the protein structure has changed [45]. The fluorescence intensity depends on whether tryptophan is in the hydrophobic environment of the core as the protein folds or is exposed to the surface as the protein unfolds. When the treatment time is 4 min, as presented in Figure 5B, compared with the fluorescence intensity of 1026 ± 9.83 of the control group (0 kV), the fluorescence intensity of trypsin increased and slightly red-shifted with the increase of ACP treatment voltage, and the fluorescence intensity reached the maximum of 1356 ± 25.18 at 50 kV. The reason for this result may be that the conformation of trypsin was changed to some extent by ACP treatment, the polypeptide was unfolded, and the aromatic amino acids were exposed to a more hydrophilic environment, thus increasing the fluorescence intensity. A strong change in the tertiary structure of proteolytic enzyme α chymotrypsin was observed after cold atmospheric pressure plasma treatment, where a significant increase in the intensity of fluorescence occurred after ACP treatment, which proves the denaturation of protein by ACP [46].

### 3.9. Effect of ACP on the Particle Size Distribution of Trypsin

The particle size distribution of protein can reflect the degradation and aggregation degree of protein [47]. Therefore, the change in particle size can also reflect the influence of ACP treatment on the structure of trypsin-like protease to some extent. When the treatment time is 4 min, the average particle size of the trypsin under different ACP treatment voltages is shown in Figure 5C. Compared with the control group (0 kV), the particle size of the sample became smaller, the particle size distribution peaks all moved towards the small particle peak with the increase of voltage (0–30 kV), and the particle size value corresponding to the peak reached the minimum of 164.2 nm; however, as the voltage was further increased to 40 kV and 50 kV, the particle size distribution shifted to the direction of large particle size. The reason may be that in the process of ACP treatment, protein particles were broken, polypeptides were unfolded, and molecules were unrolled, so the average particle size decreased. However, with the increase of voltage, the exposed surface hydrophobic groups interacted with each other, leading to the formation of trypsin-like protease aggregates, which increased the average particle size [48]. Ekezie, et al. [49] found that the average particle size of tropomyosin (TM) decreased after treatment with cold argon plasma jet (CAPJ) for 6 min, while after 15 min of treatment time the particle size increased.

### 3.10. The Effect of ACP on the SDS-PAGE of Trypsin

**Figure 6:**
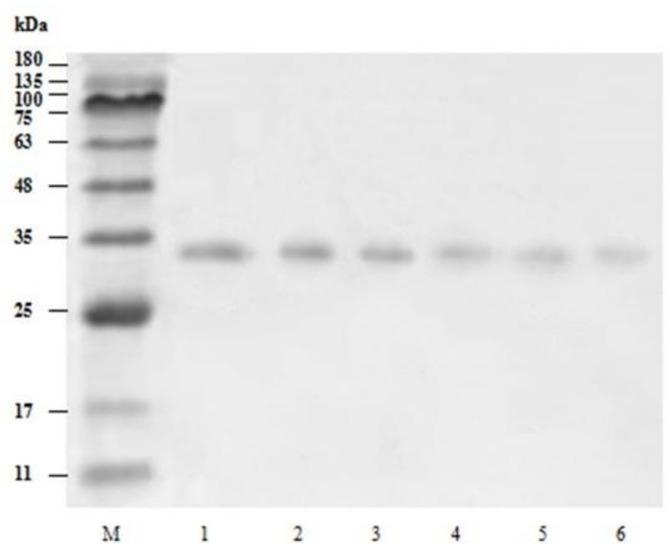
Changes in the molecular weight of trypsin after exposure to ACP at different voltages, M stands for Marker (molecular weight of standard). Lanes: 1, 0 kV; 2, 10 kV; 3, 20 kV; 4, 30 kV; 5, 40 kV; 6, 50 kV.

The SDS–PAGE was used to detect the effect of ACP treatment on trypsin structure. Protein oxidation can induce molecular structure changes in protein, including covalent cross-linking between proteins and the formation of protein fragments [50]. When the treatment time is 4 min, the results of SDS-PAGE patterns of trypsin solutions are presented in Figure 6. The findings showed that trypsin band intensity decreased with the increase of ACP treatment voltage. The decrease in the protein band intensity was significantly at the treatment voltage of 40 kV and 50 kV, proposing that these treatment voltages encourage the plasma reactive species to induce more changes in the trypsin structure. Setsuhara et al. [51] found that the etching of plasma surface by reactive species causes molecular degradation of amino acids. The decrease in protein band intensity may be due to the reaction between active oxidizing substances produced by ACP and protein, leading to the oxidative degradation of trypsin [38].

The influence of the hydroxyl radical oxidation system on the protein that oxidizes substances such as reactive oxygen species and active nitrogen was studied, and the results indicated that the oxidation system led to the reduction of the intensity of the protein band, which is consistent with our results [52]. Moreover, a strong decrease in the intensity of myosin heavy chain (MHC) bands of myofibrillar protein from *Alaska pollock* was found with the increase of ACP treatment voltages [12]. Additionally, Zhang, et al. [53] pointed out that active substances such as reactive oxygen species and active nitrogen can cause the oxidation of amino acid side chains and protein main chains, which leads to the degradation or cross-linking of the Lactate Dehydrogenase (LDH) enzyme.

### 3.11. Effect of ACP on the Secondary Structure of Trypsin

**Figure 7:**
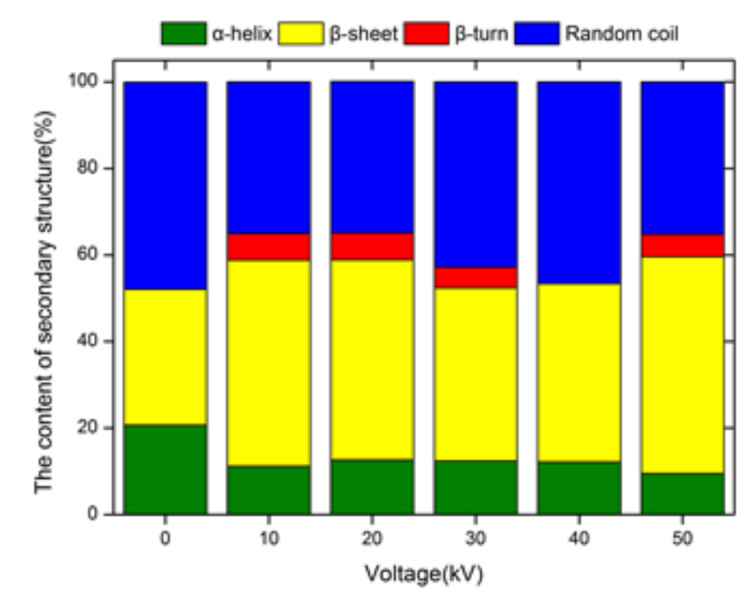
Effect of different ACP treatment voltage on the secondary structure of trypsin, 0 kV refers to control samples without ACP treatment.

The circular dichroism (CD) method is commonly used to estimate changes occurring in secondary structures of protein. When the treatment time is 4 min, the changes that occurred in the secondary structure of trypsin samples after ACP treatment is shown in Figure 7 and Table 5. The CD analysis at 180–260 nm spectra range indicates that all the ACP treated trypsin samples showed a different pattern for α-helix, β-sheet, β-turn, and random coil.

A reduction in α-helix and random coil contents was observed in the trypsin samples treated with ACP, while the contents of β-sheet and β-turn increased. The variation in the contents of α-helix, β-sheet, β-turn, and random coils reflect a considerable loss and rearrangement of structures. This indicates that the protein was significantly modified after ACP treatment. In the secondary structure of the enzyme, α-helix and β-sheet are the skeleton structures that maintain their spatial structure and hydrogen bonds that connect amino acids in the secondary structure, which leads to certain stability of the regular secondary structure. Hydrogen bonding in β-turn and random coil has a weak effect, allowing greater freedom between the residues in the peptide segment, thus showing great flexibility [54]. Therefore, the results of changes in the secondary structure of trypsin samples indicated that the secondary structure of trypsin samples tended to be disordered after ACP treatment. However, different from the overall changing trend, the content of α-helix increased slightly when the treatment voltage was 20 kV, the content of random coil increased slightly when the treatment voltage was 30 kV, and the content of β-turn decreased slightly when the treatment voltage was 40 kV. This may be because there are dipole moments, such as α-helix, which are susceptible to the electric field, and ACP treatment acting on the dipole moments causes the enzyme molecules to refold and converts the mass fraction of each unit in the secondary structure. The effects of ACP on trypsin solution secondary structure could be initiated by the oxide reactive species contained in the plasma. The results obtained in our study are consistent with the findings reported by Surowsky et al. [55] who indicated a near degradation in the secondary structure of horse-radish peroxidase after the plasma treatment time of 360 s. Moreover, similar findings on the variations in protein secondary structure have been reported in the studies conducted in gamma-irradiated milk [56], polyphenol oxidase (PPO), and peroxidase (POD) [55], and alkaline phosphatase [38] after being treated with different types of plasma equipment. However, the variations in the effects of the structure are shown to depend on the composition of the discharged gas [57]. According to the aforementioned studies, the change in the secondary structure of trypsin induced by ACP is probably attributed to the chemical reactions between the plasma species and protein resulting in the oxidization of amino acids residues causing molecular unfolding in the trypsin proteins, cleavage of peptide bonds as well as the creation of protein-protein cross-linkages.

## 4. Conclusions

The impact of ACP treatment at different times (1, 2, 3, 4 min) and voltages (10, 20, 30, 40, 50 kV) on the biochemical properties and activity of trypsin-like protease obtained from shrimp has been studied. Our findings indicated that ACP could efficiently inhibit the trypsin activity of *Litopenaeus vannamei*, and the lowest enzyme activity was achieved after being treated with ACP at 50 kV for 4 min. Moreover, the results showed a positive relation between increasing ACP treatment voltage and time, and the changes in protein secondary structure, particle size, surface hydrophobicity, as well as fluorescence intensity. Findings in the current study present a substantial understanding of how ACP plasma reactive species interacts with trypsin solution to modify the protein structures. In sum, ACP can be used as an alternative approach to inhibit the trypsin activity of *Litopenaeus vannamei*, which delays the muscle autolysis, consequently maintaining its quality and extending its shelf-life.

## Figures and Tables

**Table 1:** Summary of trypsin purification from the pancreas of *Litopenaeus vannamei*.

Purification Step	Total Activity (units)	Total Protein (mg)	Specific Activity (Units/mg Protein)	Purity (Fold)	Yield (%)
Crude enzyme	1876.3	5922.4	0.3	1.0	100
Ammonium sulfate	1544.6	512.1	3.0	10.0	82.3
Q-Sepharose Fast Flow	468.05	18.5	25.3	84.3	24.9

**Table 2:** Trypsin kinetic constants after ACP treatment.

Treatment Time (min)		Kinetic Constants	
	K_m_ (mM)	k_cat_ (s^−1^)	k_cat_/K_m_ (s^−1^ mM^−1^)
0	4.16 ± 0.01 ^d^	25.64 ± 0.16 ^a^	6.16 ± 0.08 ^a^
1	4.65 ± 0.23 ^c^	25.34 ± 0.14 ^b^	5.45 ± 0.12 ^b^
2	4.96 ± 0.08 ^b^	23.67 ± 0.18 ^c^	4.77 ± 0.15 ^c^
3	4.97 ± 0.14 ^b^	21.15 ± 0.28 ^d^	4.26 ± 0.27 ^d^
4	5.04 ± 0.20 ^a^	18.75 ± 0.09 ^e^	3.72 ± 0.19 ^e^

Values were given in terms of mean ± SD, *n* = 3. Different superscripted letters in the same column indicate a significant difference at *p* < 0.05. Zero min refers to control samples without cold plasma treatment.

**Table 3:** Effect of ACP on pH of purified trypsin at different treatment times and voltage.

Treatment Time (min)	Treatment Voltage (kV)				
	10	20	30	40	50
0	8.563 ± 0.006 ^b^	8.563 ± 0.006 ^b^	8.563 ± 0.006 ^b^	8.567 ± 0.021 ^b^	8.563 ± 0.006 ^b^
1	8.517 ± 0.015 ^b^	8.533 ± 0.006 ^b^	8.527 ± 0.006 ^b^	8.517 ± 0.006 ^b^	8.523 ± 0.006 ^b^
2	8.523 ± 0.021 ^ab^	8.527 ± 0.006 ^b^	8.533 ± 0.021 ^b^	8.503 ± 0.015 ^b^	8.513 ± 0.021 ^b^
3	8.527 ± 0.015 ^ab^	8.527 ± 0.006 ^b^	8.533 ± 0.021 ^b^	8.503 ± 0.015 ^b^	8.503 ± 0.015 ^b^
4	8.563 ± 0.032 ^a^	8.540 ± 0.026 ^ab^	8.543 ± 0.006 ^ab^	8.527 ± 0.006 ^ab^	8.523 ± 0.015 ^b^

Values were given in terms of mean ± SD, *n* = 3. Different superscripted letters in the same column indicate a significant difference at *p* < 0.05. Zero min refers to control samples without cold plasma treatment.

**Table 4:** Effect of ACP on total sulfhydryl of purified trypsin.

Treatment Time (min)	Treatment Voltage (kV)				
	10	20	30	40	50
0	0.011 ± 0.001 ^b^	0.011 ± 0.001 ^b^	0.011 ± 0.001 ^b^	0.012 ± 0.003 ^b^	0.011 ± 0.001 ^b^
1	0.012 ± 0.003 ^b^	0.012 ± 0.001 ^ab^	0.013 ± 0.003 ^a^	0.012 ± 0.001 ^b^	0.011 ± 0.001 ^b^
2	0.013 ± 0.003 ^ab^	0.013 ± 0.003 ^ab^	0.011 ± 0.001 ^b^	0.011 ± 0.004 ^b^	0.012 ± 0.001 ^ab^
3	0.013 ± 0.003 ^ab^	0.014 ± 0.004 ^a^	0.013 ± 0.000 ^a^	0.013 ± 0.003 ^a^	0.014 ± 0.003 ^a^
4	0.015 ± 0.004 ^a^	0.012 ± 0.001 ^ab^	0.012 ± 0.001 ^b^	0.013 ± 0.003 ^a^	0.014 ± 0.003 ^a^

Values were given as a mean ± standard deviation (*n* = 3). Different superscripted letters in the same column indicate a significant difference at *p* < 0.05. Zero min refers to control samples without ACP treatment.

**Table 5:** Effect of different ACP treatment voltage on the secondary structure of trypsin.

Treatment Voltage (kV)	Secondary Structure of Protein (%)			
	α-Helix	β-Sheet	β-Turn	Random Coil
0	20.70	31.40	0.00	47.80
10	11.20	47.60	6.20	35.00
20	12.70	46.20	6.20	35.00
30	12.40	40.00	4.80	42.80
40	12.20	41.20	0.00	46.60
50	9.50	50.10	5.20	35.20

## Data Availability

The data presented in this study are available on request from the corresponding author.

## References

[1] Singh A., Benjakul S. (2018). Proteolysis and its control using protease inhibitors in fish and fish products: A review. Compr. Rev. Food Sci. Food Saf..

[2] Singh J., Singh B. (2020). Inhibition of post-mortem fish muscle softening and degradation using legume seed proteinase inhibitors. J. Food Sci. Technol..

[3] Weiss F.U., Halangk W., Lerch M.M. (2008). New advances in pancreatic cell physiology and pathophysiology. Best Pract. Res. Clin. Gastroenterol..

[4] Brauer J., Salazar-Leyva J., Bringas L., Rouzaud-Sández O. (2003). Effect of dietary protein on muscle collagen, collagenase and shear force of farmed white shrimp (*Litopenaeus vannamei*). Eur. Food Res. Technol..

[5] Sriket C., Benjakul S., Visessanguan W. (2011). Characterisation of proteolytic enzymes from muscle and hepatopancreas of fresh water prawn (*Macrobrachium rosenbergii*). J. Sci. Food Agric..

[6] Senphan T., Benjakul S. (2012). Compositions and yield of lipids extracted from hepatopancreas of Pacific white shrimp (*Litopenaeus vannamei*) as affected by prior autolysis. Food Chem..

[7] Cao W., Tan C., Zhan X., Li H., Zhang C. (2014). Ultraviolet irradiation and gradient temperature assisted autolysis for protein recovery from shrimp head waste. Food Chem..

[8] Xiao H., Lin Q., Li Y., Zhao M. (2004). Advances on applied studies of protease inhibitor in gene engineering. Biotechnol. Bull..

[9] Bijina B., Chellappan S., Krishna J.G., Basheer S.M., Elyas K.K., Bahkali A.H., Chandrasekaran M. (2011). Protease inhibitor from Moringa oleifera with potential for use as therapeutic drug and as seafood preservative. Saudi J. Biol. Sci..

[10] Klomklao S., Benjakul S. (2015). Effect of trypsin inhibitor in adzuki bean (*Vigna angularis*) on proteolysis and gel properties of threadfin bream (*Nemipterus bleekeri*). LWT Food Sci. Technol..

[11] Garcia-Galan C., Berenguer-Murcia A., Fernandez-Lafuente R., Rodrigues R. (2012). Potential of different enzyme immobilization strategies to improve enzyme performance. Adv. Synth. Catal..

[12] Miao W.H., Nyaisaba B.M., Koddy J.K., Chen M.L., Hatab S., Deng S.G. (2020). Effect of cold atmospheric plasma on the physicochemical and functional properties of myofibrillar protein from Alaska pollock (*Theragra chalcogramma*). Int. J. Food Sci. Technol..

[13] Misra N.N., Jo C. (2017). Applications of cold plasma technology for microbiological safety in meat industry. Trends Food Sci. Technol..

[14] Bußler S., Ehlbeck J., Schlüter O. (2016). Pre-drying treatment of plant related tissues using plasma processed air: Impact on enzyme activity and quality attributes of cut apple and potato. Innov. Food Sci. Emerg..

[15] Pankaj S.K., Misra N.N., Cullen P.J. (2013). Kinetics of tomato peroxidase inactivation by atmospheric pressure cold plasma based on dielectric barrier discharge. Innov. Food Sci. Emerg..

[16] Nyaisaba B.M., Miao W.H., Hatab S., Siloam A., Chen M.L., Deng S.G. (2019). Effects of cold atmospheric plasma on squid proteases and gel properties of protein concentrate from squid (*Argentinus ilex*) mantle. Food Chem..

[17] Khantaphant S., Benjakul S. (2010). Purification and characterization of trypsin from the pyloric caeca of brownstripe red snapper (*Lutjanus vitta*). Food Chem..

[18] Kristjansson M.M. (1991). Purification and characterization of trypsin from the pyloric caeca of rainbow trout (*Oncorhynchus mykiss*). J. Agric. Food Chem..

[19] Lowry O., Rosebrough N., Farr A.L., Randall R. (1951). Protein measurement with the folin reagent. J. Biol. Chem..

[20] Laemmli U.K. (1970). Cleavage of structural proteins during the assembly of the head of bacteriophage T4. Nature.

[21] Yoshida A., Bae I., Sonoda H., Masuo R., Oda S., Cao M.J., Osatomi K., Hara K. (2009). Characterization of gelatinolytic enzymes in the skeletal muscle of red sea bream pagrus major. Fish. Sci..

[22] Cao Y.G., Zhao J., Xiong Y.L.L. (2016). Coomassie brilliant blue-binding: A simple and effective method for the determination of water-insoluble protein surface hydrophobicity. Anal. Methods.

[23] Nikoo M., Benjakul S., Xu X.M. (2015). Antioxidant and cryoprotective effects of Amur sturgeon skin gelatin hydrolysate in unwashed fish mince. Food Chem..

[24] Farasat M., Arjmand S., Siadat S.O.R., Sefidbakht Y., Ghomi H. (2018). The effect of non-thermal atmospheric plasma on the production and activity of recombinant phytase enzyme. Sci. Rep..

[25] Li Y., Zhong F., Ji W., Yokoyama W., Shoemaker C.F., Zhu S., Xia W.S. (2013). Functional properties of maillard reaction products of rice protein hydrolysates with mono-, oligo- and polysaccharides. Food Hydrocolloid.

[26] Zhang H., Li L., Tatsumi E., Isobe S. (2004). High-pressure treatment effects on proteins in soy milk. LWT—Food Sci. Technol..

[27] Wang Y., Liu M., Zhao L., Qiu Y., Zhuang Y. (2015). Influence of processing conditions on reducing γ-aminobutyric acid content during fortified milk production. Food Res. Int..

[28] Jones R.G., Landon J. (2002). Enhanced pepsin digestion: A novel process for purifying antibody F(ab’)(2) fragments in high yield from serum. J. Immunol. Methods.

[29] Senphan T., Benjakul S., Kishimura H. (2015). Purification and characterization of trypsin from hepatopancreas of Pacific white shrimp. J. Food Biochem..

[30] Poonsin T., Simpson B.K., Benjakul S., Visessanguan W., Yoshida A., Osatomi K., Klomklao S. (2019). Anionic trypsin from the spleen of albacore tuna (*Thunnus alalunga*): Purification, biochemical properties and its application for proteolytic degradation of fish muscle. Int. J. Biol. Macromol..

[31] Honjo I., Kimura S., Nonaka M. (1990). Purification and characterization of trypsin-like enzyme from shrimp *Penaeus indicus*. Nippon Suisan Gakk.

[32] Perera E., Rodríguez-Viera L., Perdomo-Morales R., Montero-Alejo V., Moyano F.J., Martínez-Rodríguez G., Mancera J.M. (2015). Trypsin isozymes in the lobster *Panulirus argus* (Latreille, 1804): From molecules to physiology. J. Comp. Physiol. B.

[33] Zhang Q.Z., Cheng Z.Z., Zhang J.H., Nasiru M.M., Wang Y.B., Fu L.L. (2021). Atmospheric cold plasma treatment of soybean protein isolate: Insights into the structural, physicochemical, and allergenic characteristics. J. Food Sci..

[34] Choi S., Attri P., Lee I., Oh J., Yun J.H., Park J., Choi E., Lee W. (2017). Structural and functional analysis of lysozyme after treatment with dielectric barrier discharge plasma and atmospheric pressure plasma jet. Sci. Rep..

[35] Li H.P., Wang L.Y., Li G., Jin L., Le P.S., Zhao H.X., Xing X.H., Bao C.Y. (2011). Manipulation of lipase activity by the helium radio-frequency, atmospheric-pressure glow discharge plasma jet. Plasma Processes Polym..

[36] Ali A., Ashraf Z., Kumar N., Rafiq M., Jabeen F., Park J., Choi K., Lee S., Seo S.-Y., Choi E. (2016). Influence of plasma-activated compounds on melanogenesis and tyrosinase activity. Sci. Rep..

[37] Puač N., Škoro N., Spasic K., Živković S., Milutinovic M., Malovic G., Petrović Z. (2017). Activity of catalase enzyme in Paulownia tomentosa seeds during the process of germination after treatments with low pressure plasma and plasma activated water. Plasma Processes Polym..

[38] Segat A., Misra N.N., Cullen P.J., Innocente N. (2016). Effect of atmospheric pressure cold plasma (ACP) on activity and structure of alkaline phosphatase. Food Bioprod. Process..

[39] Ji Y.B., Wang F.L. (2021). Optimization of trypsin extraction technology of Allium cepa L. polysaccharide by response surface methodology and the antitumor effects through immunomodulation. Bioengineered.

[40] Meinlschmidt P., Ueberham E., Lehmann J., Reineke K., Schluter O., Schweiggert-Weisz U., Eisner P. (2016). The effects of pulsed ultraviolet light, cold atmospheric pressure plasma, and gamma-irradiation on the immunoreactivity of soy protein isolate. Innov. Food Sci. Emerg..

[41] Benjakul S., Morrissey M.T. (1997). Protein hydrolysates for Pacific whiting solid wastes. J. Agric. Food Chem..

[42] Liu Z.W., Manzoor M.F., Tan Y.C., Inam-ur-Raheem M., Aadil R.M. (2020). Effect of dielectric barrier discharge (DBD) plasma on the structure and antioxidant activity of bovine serum albumin (BSA). Int. J. Food Sci. Technol..

[43] Xie Y., Chen B., Guo J., Nie W., Zhou H., Li P.J., Zhou K., Xu B.C. (2021). Effects of low voltage electrostatic field on the microstructural damage and protein structural changes in prepared beef steak during the freezing process. Meat Sci..

[44] Sun N.K., Lee S., Bin Song K. (2002). Effect of high-pressure treatment on the molecular properties of mushroom polyphenoloxidase. LWT-Food Sci. Technol..

[45] Zhou L.Y., Wu J.H., Hu X.S., Zhi X., Liao X.J. (2009). Alterations in the activity and structure of pectin methylesterase treated by high pressure carbon dioxide. J. Agric. Food Chem..

[46] Attri P., Choi E. (2013). Influence of reactive oxygen species on the enzyme stability and activity in the presence of ionic liquids. PLoS ONE.

[47] Schmitt C., Bovay C., Rouvet M., Shojaei-Rami S., Kolodziejczyk E. (2007). Whey protein soluble aggregates from heating with NaCl: Physicochemical, interfacial, and foaming properties. Langmuir.

[48] Rastogi N.K., Raghavarao K., Balasubramaniam V.M., Niranjan K., Knorr D. (2007). Opportunities and challenges in high pressure processing of foods. Crit. Rev. Food Sci..

[49] Ekezie F.C., Sun D.W., Cheng J.H. (2019). Altering the IgE binding capacity of king prawn (*Litopenaeus Vannamei*) tropomyosin through conformational changes induced by cold argon-plasma jet. Food Chem..

[50] Fang M.X., Luo X.Y., Xiong S.B., Yin T., Hu Y., Liu R., Du H.Y., Liu Y.M., You J. (2021). In vitro trypsin digestion and identification of possible cross-linking sites induced by transglutaminase (TGase) of silver carp (*Hypophthalmichthys molitrix*) surimi gels with different degrees of cross-linking. Food Chem..

[51] Setsuhara Y., Cho K., Shiratani M., Sekine M., Hori M. (2013). Plasma interactions with aminoacid (l-alanine) as a basis of fundamental processes in plasma medicine. Curr. Appl. Phys..

[52] Priya Arjunan K., Morss Clyne A. (2011). Hydroxyl radical and hydrogen peroxide are primarily responsible for dielectric barrier discharge plasma-induced angiogenesis. Plasma Processes Polym..

[53] Zhang H., Xu Z., Shen J., Li X., Ding L., Ma J., Lan Y., Xia W., Cheng C., Sun Q. (2015). Effects and mechanism of atmospheric-pressure dielectric barrier discharge cold plasmaon *Lactate Dehydrogenase* (LDH) Enzyme. Sci. Rep..

[54] Kikuchi H., Wako H., Yura K., Go M., Mimuro M. (2000). Significance of a two-domain structure in subunits of phycobiliproteins revealed by the normal mode analysis. Biophys. J..

[55] Surowsky B., Fischer A., Schlueter O., Knorr D. (2013). Cold plasma effects on enzyme activity in a model food system. Innov. Food Sci. Emerg..

[56] Huang M., Li X., Tong P., Gao J., Yuan J., Yang A., Chen H., Wu Y. (2020). Potential allergenicity assessment after bovine apo-α-actalbumin binding to calcium ion. J. Food Biochem..

[57] Attri P., Kumar N., Park J.H., Yadav D.K., Choi S., Uhm H.S., Kim I.T., Choi E.H., Lee W. (2015). Influence of reactive species on the modification of biomolecules generated from the soft plasma. Sci. Rep..

